# Childhood asthma is associated with COPD and known asthma variants in COPDGene: a genome-wide association study

**DOI:** 10.1186/s12931-018-0890-0

**Published:** 2018-10-29

**Authors:** Lystra P. Hayden, Michael H. Cho, Benjamin A. Raby, Terri H. Beaty, Edwin K. Silverman, Craig P. Hersh, on behalf of the COPDGene Investigators, James D. Crapo, Edwin K. Silverman, Barry J. Make, Elizabeth A. Regan, Terri Beaty, Ferdouse Begum, Peter J. Castaldi, Michael Cho, Dawn L. DeMeo, Adel R. Boueiz, Marilyn G. Foreman, Eitan Halper-Stromberg, Lystra P. Hayden, Craig P. Hersh, Jacqueline Hetmanski, Brian D. Hobbs, John E. Hokanson, Nan Laird, Christoph Lange, Sharon M. Lutz, Merry-Lynn McDonald, Margaret M. Parker, Dandi Qiao, Elizabeth A. Regan, Edwin K. Silverman, Emily S. Wan, Sungho Won, Phuwanat Sakornsakolpat, Dmitry Prokopenko, Mustafa Al Qaisi, Harvey O. Coxson, Teresa Gray, Mei Lan K. Han, Eric A. Hoffman, Stephen Humphries, Francine L. Jacobson, Philip F. Judy, Ella A. Kazerooni, Alex Kluiber, David A. Lynch, John D. Newell, Elizabeth A. Regan, James C. Ross, Raul San Jose Estepar, Joyce Schroeder, Jered Sieren, Douglas Stinson, Berend C. Stoel, Juerg Tschirren, Edwin Van Beek, Bram van Ginneken, Eva van Rikxoort, George Washko, Carla G. Wilson, Robert Jensen, Douglas Everett, Jim Crooks, Camille Moore, Matt Strand, Carla G. Wilson, John E. Hokanson, John Hughes, Gregory Kinney, Sharon M. Lutz, Katherine Pratte, Kendra A. Young, Surya Bhatt, Jessica Bon, Mei Lan K. Han, Barry Make, Carlos Martinez, Susan Murray, Elizabeth Regan, Xavier Soler, Carla G. Wilson, Russell P. Bowler, Katerina Kechris, Farnoush Banaei-Kashani, Jeffrey L. Curtis, Carlos H. Martinez, Perry G. Pernicano, Nicola Hanania, Philip Alapat, Mustafa Atik, Venkata Bandi, Aladin Boriek, Kalpatha Guntupalli, Elizabeth Guy, Arun Nachiappan, Amit Parulekar, Dawn L. DeMeo, Craig Hersh, Francine L. Jacobson, George Washko, Graham Barr, John Austin, Belinda D’Souza, Gregory D. N. Pearson, Anna Rozenshtein, Byron Thomashow, Neil MacIntyre, Page McAdams, Lacey Washington, Charlene McEvoy, Joseph Tashjian, Robert Wise, Robert Brown, Nadia N. Hansel, Karen Horton, Allison Lambert, Nirupama Putcha, Richard Casaburi, Alessandra Adami, Matthew Budoff, Hans Fischer, Janos Porszasz, Harry Rossiter, William Stringer, Michael E. DeBakey, Amir Sharafkhaneh, Charlie Lan, Christine Wendt, Brian Bell, Marilyn G. Foreman, Eugene Berkowitz, Gloria Westney, Russell Bowler, David A. Lynch, Richard Rosiello, David Pace, Gerard Criner, David Ciccolella, Francis Cordova, Chandra Dass, Gilbert D’Alonzo, Parag Desai, Michael Jacobs, Steven Kelsen, Victor Kim, James Mamary, Nathaniel Marchetti, Aditi Satti, Kartik Shenoy, Alex Swift, Irene Swift, Maria Elena Vega-Sanchez, Mark Dransfield, William Bailey, Surya Bhatt, Anand Iyer, Hrudaya Nath, J. Michael Wells, Joe Ramsdell, Paul Friedman, Xavier Soler, Andrew Yen, Alejandro P. Comellas, Karin F. Hoth, John Newell, Brad Thompson, Mei Lan K. Han, Ella Kazerooni, Carlos H. Martinez, Joanne Billings, Abbie Begnaud, Tadashi Allen, Frank Sciurba, Jessica Bon, Divay Chandra, Carl Fuhrman, Joel Weissfeld, Antonio Anzueto, Sandra Adams, Diego Maselli-Caceres, Mario E. Ruiz

**Affiliations:** 1 Division of Respiratory Diseases, Boston Children’s Hospital, Boston, MA USA; 2 Channing Division of Network Medicine, Brigham and Women’s Hospital, 181 Longwood Avenue, Boston, MA 02115 USA; 3 Division of Pulmonary and Critical Care Medicine, Brigham and Women’s Hospital, Boston, MA USA; 4 Bloomberg School of Public Health, Johns Hopkins University, Baltimore, MD USA

**Keywords:** Childhood asthma, Genome-wide association study, Chronic obstructive pulmonary disease, Lung function, Genetic epidemiology

## Abstract

**Background:**

Childhood asthma is strongly influenced by genetics and is a risk factor for reduced lung function and chronic obstructive pulmonary disease (COPD) in adults. This study investigates self-reported childhood asthma in adult smokers from the COPDGene Study. We hypothesize that childhood asthma is associated with decreased lung function, increased risk for COPD, and that a genome-wide association study (GWAS) will show association with established asthma variants.

**Methods:**

We evaluated current and former smokers ages 45–80 of non-Hispanic white (NHW) or African American (AA) race. Childhood asthma was defined by self-report of asthma, diagnosed by a medical professional, with onset at < 16 years or during childhood. Subjects with a history of childhood asthma were compared to those who never had asthma based on lung function, development of COPD, and genetic variation. GWAS was performed in NHW and AA populations, and combined in meta-analysis. Two sets of established asthma SNPs from published literature were examined for association with childhood asthma.

**Results:**

Among 10,199 adult smokers, 730 (7%) reported childhood asthma and 7493 (73%) reported no history of asthma. Childhood asthmatics had reduced lung function and increased risk for COPD (OR 3.42, 95% CI 2.81–4.18). Genotype data was assessed for 8031 subjects. Among NHWs, 391(7%) had childhood asthma, and GWAS identified one genome-wide significant association in *KIAA1958* (rs59289606, *p* = 4.82 × 10^− 8^). Among AAs, 339 (12%) had childhood asthma. No SNPs reached genome-wide significance in the AAs or in the meta-analysis combining NHW and AA subjects; however, potential regions of interest were identified. Established asthma SNPs were examined, seven from the NHGRI-EBI database and five with genome-wide significance in the largest pediatric asthma GWAS. Associations were found in the current childhood asthma GWAS with known asthma loci in *IL1RL1*, *IL13*, *LINC01149*, near *GSDMB*, and in the *C11orf30-LRRC32* region (Bonferroni adjusted *p* < 0.05 for all comparisons).

**Conclusions:**

Childhood asthmatics are at increased risk for COPD. Defining asthma by self-report is valid in populations at risk for COPD, identifying subjects with clinical and genetic characteristics known to associate with childhood asthma. This has potential to improve clinical understanding of asthma-COPD overlap (ACO) and enhance future research into ACO-specific treatment regimens.

**Trial registration:**

ClinicalTrials.gov, NCT00608764 (Active since January 28, 2008).

**Electronic supplementary material:**

The online version of this article (10.1186/s12931-018-0890-0) contains supplementary material, which is available to authorized users.

## Background

Asthma is the most common chronic disease of childhood, affecting 8.4% of children in the United States [1]. Childhood asthma is a genetically distinct asthma subtype, with estimated heritability of 68–92% [2–10]. Asthma is a known risk factor for development of reduced lung function and chronic obstructive pulmonary disease (COPD) in adults [11–14]. Asthmatics are often excluded from COPD studies, and thus information on the mechanism of disease and appropriate treatments for asthma-COPD overlap (ACO) remains limited [15].

We have previously examined self-reported history of childhood asthma and ACO in the COPDGene Study, a cohort of more than 10,000 adult smokers with and without COPD. We have shown that a combined history of childhood pneumonia with childhood asthma is a risk factor for developing COPD [16]. We demonstrated that that ACO subjects were younger with a lower lifetime smoking intensity, and that both childhood asthmatics and ACO subjects have increased airways disease on chest computed tomography scan [17–19]. Longitudinal analysis has shown that early-life asthmatics do not have increased rates of lung function decline despite their increased exacerbation frequency [20]. We have not previously examined childhood asthma as an independent risk factor for COPD in this population.

As interest in the long-term outcomes of early-life asthmatics has expanded, a recurring question has risen about the validity of using self-reported asthma history in large adult cohorts recruited for genetic epidemiology studies [21, 22]. This has been particularly important when trying to understand Asthma-COPD Overlap (ACO), as epidemiologic studies of COPD, an adult disease, rely on self-reported history of asthma early in life both for exclusion criteria and for analysis [15, 23]. Our current investigation examines self-reported childhood asthma in adult smokers from the COPDGene Study, to see if they share similar phenotypes and genotypes with those seen in asthmatic subjects from prior studies, including published GWAS [2, 3, 24]. Prior GWAS have implicated genetic susceptibility specific to childhood asthma, including variants in *ORMDL3/GSDMB*, *IL1RL1*, *IL33*, and *RAD50* [4, 7, 24]. We hypothesize that self-reported history of childhood asthma will be associated with decreased lung function and increased risk for COPD in adult smokers, and that a genome-wide association study (GWAS) will show an increased prevalence of known asthma variants among childhood asthmatics, compared to those subjects who never had asthma.

## Methods

### Subjects

We evaluated 10,199 current and former adult smokers enrolled in the COPDGene Study between 2008 and 2011 (August 31, 2016 dataset). The COPDGene Study is a multicenter, observational study designed to identify genetic and environmental factors associated with COPD. Subjects included were of non-Hispanic (NHW) white or African American (AA) race, between 45 and 80 years of age, and had at least a 10 pack-year history of smoking. We excluded subjects with lung disease other than asthma or COPD, or those who were nonsmokers. Subjects were recruited at 21 clinical sites within the U.S. Each site received institutional review board approval and each participant provided informed consent [25]. Details of the study protocol and data forms are available at www.copdgene.org [25, 26].

### Data collection

Subject responses to a modified American Thoracic Society Respiratory Epidemiology Questionnaire were used to collect asthma history [26, 27]. A standardized spirometry protocol pre and post-albuterol was completed (ndd EasyOne Spirometer, Zurich, Switzerland). DNA extracted from blood samples was genotyped using HumanOmniExpress arrays (Illumina, San Diego, CA). DNA and single nucleotide polymorphism (SNP) data underwent standard quality control measures [25, 28, 29]. Imputation used 1000 Genomes Phase I v3 reference panels (hg19) to obtain additional genotypes with MaCH and minimac (exomeChip pipelineV1.4) [29–32]. Imputation quality was assessed using Rsq, and SNPs with Rsq > 0.3 were considered to be of acceptable quality. COPDGene datasets are publicly available (dbGaP accession number phs000179.v1.p1).

### Case identification

Asthma history was assessed by questionnaire response. Subjects were asked if they had ever had asthma, at what age it started, and if it was diagnosed by a doctor or other health professional. Childhood asthma was defined as self-report of asthma diagnosed by a health professional with age of onset at < 16 years or as a child with exact age not known [5, 16, 20, 33]. Subjects were classified as never having asthma if they responded “No” to asthma history on the questionnaire. As in previous COPDGene publications, COPD was defined as Global Initiative for Chronic Obstructive Lung Disease (GOLD) 2007 spirometry grades 2–4, corresponding to post-bronchodilator forced expiratory volume in the first second (FEV_1_) to forced vital capacity (FVC) ratio < 0.7 with FEV_1_ < 80% predicted [16–18, 20, 34]. Control smokers had normal spirometry, defined as FEV_1_/FVC ≥ 0.7 and FEV_1_ ≥ 80%.

### Statistical analysis

Subjects with childhood asthma were compared to subjects who never had asthma on measures of lung function and development of COPD. Subjects with missing or unclassifiable responses were removed from the specific analysis. Statistical analysis used R v3.1.1. Single variable analysis used chi-square tests, Wilcoxon rank sum tests, or t-tests. Multivariable regression analysis was performed, all models were adjusted for number of pack years of smoking history. COPD and FEV_1_/FVC models were additionally adjusted for gender, age at enrollment, and race. The FEV_1_/FVC model was also adjusted for height; percent predicted FEV_1_ and FVC values have these factors accounted for in the baseline variable thus no adjustment was indicated. Logistic regression reported odds ratio (OR) with 95% confidence interval (CI) and linear regression reported absolute difference (β) with standard error (SE).

GWAS were performed comparing those with history of childhood asthma to those who never had asthma using PLINK 1.90 [35]. GWAS were performed in the NHW and AA populations separately using logistic regression adjusted for sex and principal components (PC) for genetic ancestry [28]. SNPs were considered to reach genome-wide significance at the threshold of *p* ≤ 5 × 10^− 8^_,_ and regions of interest were identified at near genome-wide significance with *p* ≤ 1 × 10^− 6^ [36, 37]. The degree of genomic inflation after PC adjustment was assessed [38]. Results from the NHW and AA populations were combined in a fixed effects meta-analysis with inverse variance weighting [35, 39].

### Comparison to published asthma GWAS

The childhood asthma GWAS results were examined for association with established asthma SNPs derived from the National Human Genome Research Institute - European Bioinformatics Institute (NHGRI-EBI) Catalog of published genome-wide association studies (Sept 28, 2017 search, https://www.ebi.ac.uk/gwas/search) [2, 3]. A search for the disease/trait “asthma” identified 85 studies, with 600 SNP associations. Eight SNPs were identified in more than one NHGRI-EBI catalog study, and were considered established asthma SNPs: rs10197862 in *IL1RL1*, rs1837253 near *TSLP*, rs2244012 in *RAD50*, rs1295686 in *IL13*, rs7130588 near the *C11orf30-LRRC32* region, rs3894194 in *GSDMA,* rs2305480 and rs11078927 in *GSDMB* [7, 40–50]. Two *GSDMB* SNPs were in high linkage disequilibrium (r^2^ > 0.8). We selected the SNP that was genotyped in our population, rs2305480, for inclusion, and rs11078927 was excluded, leaving seven established asthma SNPs. These seven established asthma SNPs were examined for association with childhood asthma in the current GWAS, with a Bonferroni correction for seven tests performed, and a significance level of 0.05. Hypergeometric testing was used to calculate the likelihood that the established asthma SNPs were over-represented in this sample.

The childhood asthma GWAS results were additionally examined for association with the five genetic loci found to be associated with pediatric asthma in the largest pediatric asthma GWAS to date (8976 cases, 18,399 controls) from the Trans-National Asthma Genetic Consortium (TAGC): rs4988958 in *IL1RL1*, rs1295685 in *IL13*, rs2596464 in *LINC01149*, rs12551256 in *IL33*, rs8069176 near *GSDMB* [24]. These five TAGC pediatric asthma SNPs were examined for association with childhood asthma in the current GWAS, with a Bonferroni correction for five tests performed, and a significance level of 0.05.

## Results

### Subject classification

Of 10,199 subjects in the COPDGene study, 730 (7%) had childhood asthma and 7493 (73%) were control subjects without childhood asthma (Table 1). There were 1976 subjects removed from the analysis: 52 with responses to the childhood asthma questions that were not classifiable, 1229 who reported a history of asthma that did not start in childhood, and 695 who reported they did not know if they had asthma (Additional file 1: Figure S1).

**Table 1:** Characteristics of childhood asthma subjects compared to subjects without asthma in COPDGene

	Childhood Asthma	Never Asthma	*P*-value
	*N = 730*	*N = 7493*	
Male gender (%)^a^	388 (53%)	4255 (57%)	0.06
Mean age, year (SD)^c^	58 (9)	60 (9)	< 0.001
Non-Hispanic white (%)^a^	391 (54%)	5075 (68%)	< 0.001
African American (%)^a^	339 (46%)	2418 (32%)	< 0.001
Pack-years of smoking (SD)^b^	43 (25)	44 (25)	0.09
Current smoking (%)^a^	410 (56%)	4041 (54%)	0.26
History of hay fever (%)^a^	385 (53%)	1727 (23%)	< 0.001

Univariate analysis with ^a^ Chi-square ^b^ Wilcoxon rank sum test ^c^ t-test

### Subject characteristics

Compared to subjects without childhood asthma, those with history of childhood asthma were younger and more likely to be AA. Subjects with childhood asthma had increased odds of developing COPD (OR 3.42, 95% CI 2.81–4.18) (Table 2). When compared to those without childhood asthma, adult smokers with asthma during childhood also had reduced lung function measured by FEV_1_, FVC and FEV_1_/FVC (*p* < 0.001 for all comparisons).

**Table 2:** COPD and lung function in subjects with childhood asthma compared to never asthma subjects

	Childhood	Never	Impact of Childhood Asthma		
	Asthma	Asthma			
	*N = 730*	*N = 7493*	OR	95% CI	*P*-value^a^
COPD (%)^b,d, e^	341 (47%)	2303 (31%)	3.42	(2.81, 4.18)	< 0.001
			β	SE	*P*-value
FEV_1_ post-bronchodilator % predicted (SD)^c,d^	69% (25)	80% (26)	−10.44	0.92	< 0.001
FVC post-bronchodilator % predicted (SD)^c,d^	83% (19)	89% (18)	−6.01	0.68	< 0.001
FEV_1_/FVC post-bronchodilator (SD)^c,d,e,f^	0.64 (0.16)	0.68 (0.16)	−0.06	0.01	< 0.001

Abbreviations: *COPD* chronic obstructive pulmonary disease; *FEV*_*1*_ forced expiratory volume in the first second; *SD* standard deviation; *FVC* forced vital capacity. ^a^Each row is a separate model: ^b^Logistic regression with odds ratio (OR), 95% confidence interval (CI); ^c^Linear regression with beta coefficient (β), standard error (SE). Covariates: ^d^pack years; ^e^gender, age at enrollment, race; ^f^height

### GWAS

Genotype data was assessed in 8031 subjects who met case/control criteria (Additional file 1: Figure S1). Among the 5364 NHWs, there were 385 (7%) childhood asthma cases and 4979 (93%) controls without childhood asthma. Among the 2667 AAs, there were 325 (12%) childhood asthma cases and 2342 (88%) controls. NHW and AA GWAS included all SNPs with minor allele frequency (MAF) ≥ 5%, Quantile-Quantile (QQ) and Manhattan plots including can be viewed in Additional file 1: Figure S2 and S3. Lambda values were 1.01 in both NHW and AA populations. Meta-analysis included all SNPs with MAF ≥ 1%, with a lower MAF cutoff used to account for the more modest correlation of variants between ancestries; QQ and Manhattan plots are in Additional file 1: Figure S4 [39, 51].

**Fig. 1:**
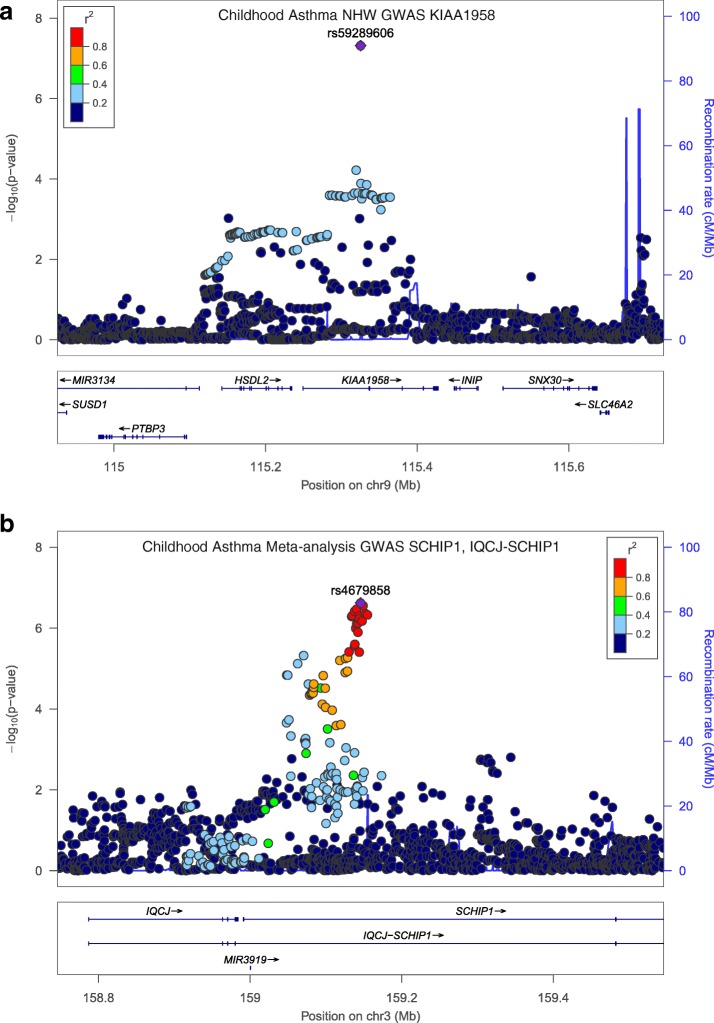
LocusZoom Plot of childhood asthma GWAS variants **a** from non-Hispanic whites in KIAA1958 and **b** from the meta-analysis in the region near SCHIP1 and its read through transcript IQCJ-SCHIP1

In the NHW childhood asthma GWAS, one SNP reached the level of genome-wide significance, rs59289606 located in the gene *KIAA1958* (*p* = 4.82 × 10^− 8^) (Table 3, Fig. 1, Additional file 1: Figure S2). An additional region of interest was identified in *PDZD2* with three SNPs approaching genome-wide significance (*p* = 3.9–7.80 × 10^− 7^) (Additional file 1: Figure S5). In the AA childhood asthma GWAS, no SNPs reached the level of genome-wide significance, however, regions of interest were identified in *FLJ12825* (*p* = 1.75 × 10^− 7^) and *PHF14* (*p* = 4.94 × 10^− 7^) (Additional file 1: Figure S6). Meta-analysis combined the NHW and AA results from this study. No meta-analysis SNPs reached the level of genome-wide significance. However, a region of interest was identified in *SCHIP1* and its read through transcript *IQCJ-SCHIP1* with 14 SNPs approaching genome-wide significance (*p* = 2.39–7.84 × 10^− 7^) (Fig. 1).

**Table 3:** Top SNPs from childhood asthma GWAS in COPDGene^e^

	**Locus**	**SNP**	**Effect Allele**	**OR**	**Rsq**	**Freq (%)**		**P-value** ^b,d^	**Gene Symbol**	**Gene Name**
**NHW** ^a^	9q32	rs59289606	A	2.06	0.38	20%		4.82E-08	*KIAA1958*	
(8,900,203 SNPs)	5p13.3	rs439399	C	1.52	0.99	22%		3.90E-07	*PDZD2*	PDZ domain containing 2
**AA** ^a^	12q13.13	rs7315121	T	2.90	0.45	7%		1.75E-07	*FLJ12825*	uncharacterized LOC440101
(15,374,350 SNPs)	7p21.3	rs56317450	A	2.11	0.79	8%		4.94E-07	*PHF14*	PHD finger protein 14
	**Locus**	**SNP**	**Effect Allele**	**OR**	**Rsq** **NHW, AA**	**Freq (%)** **NHW, AA**	**I** ^**2**^	**P-value** ^c,d^	**Gene Symbol**	**Gene Name**
**Meta-analysis** (7,658,992 SNPs)	3q25.32-q25.33	rs4679858	G	1.48	0.99, 0.98	14%, 12%	0	2.39E-07	*SCHIP1* *IQCJ-SCHIP1*	schwannomin interacting protein 1 & the readthrough

Abbreviations: *GWAS* genome-wide association study; *NHW* Non-Hispanic Whites; *AA* African American; *SNP* single nucleotide polymorphism; *OR* odds ratio of effect allele; *Rsq* estimation of imputation quality; *Freq* frequency of effect allele; *I*^*2*^ heterogeneity index; ^a^ Each population was run as an independent analysis, and then combined in the meta-analysis: ^b^ Logistic regression based on case control status; ^c^ Fixed effect meta-analysis weighted by inverse variance. ^d^ Adjusted for sex, genetic ancestry. ^e^ Includes the top SNP from each region with GWAS P-value ≤1 × 10–6

### Association testing of established asthma variants

Seven NHGRI-EBI SNPs previously known to be associated with asthma were extracted from the current GWAS results (Table 4). Among the NHW population, the childhood asthma GWAS found association with a known asthma variants in *IL1RL1* (rs10197862, adjusted *p* = 0.011) and approached significance in *GSDMB* (rs2305480, adjusted *p* = 0.060) and near the *C11orf30-LRRC32* region (rs7130588, adjusted *p* = 0.054) (Additional file 1: Figure S7). The meta-analysis of NHW and AA subjects in our childhood asthma GWAS found association with known asthma variants in *IL1RL1* (rs10197862, adjusted *p* = 0.042), in *IL13* (rs1295686, adjusted *p* = 0.043), and near the *C11orf30-LRRC32* region (rs7130588, adjusted *p* = 0.005) (Additional file 1: Figure S8). There is agreement in direction of effect in these four associated or near associated SNPs in both NHW and AA populations as well as with prior published GWAS [7, 40, 44, 45, 49, 50]. Hypergeometric testing showed that when selecting seven SNPs from the meta-analysis population, the probability of having one or more SNP significant at this level was *p* = 0.034. Among the meta-analysis population, when selecting seven SNPs, the probability of having three or more SNP significant at this level was *p* = 1.21 × 10^− 05^.

**Table 4:** Associations with NHGRI-EBI asthma SNPs in COPDGene childhood asthma GWAS

**NHW** ^a^	**Gene**	**SNP**	**Locus**	**Effect Allele**	**OR**	**Rsq**	**Freq (%)**	**P-value** ^b,d^	**Adj P-value** ^e^
*(385 cases, 4979 controls)*	***IL1RL1***	**rs10197862** ^**f**^	**2q12.1**	**A**	**1.45**	**1.00**	0.85	**0.002**	**0.011**
	*TSLP*	rs1837253^g^	5q22.1	T	1.07		0.75	0.414	1.000
	*RAD50*	rs2244012^f^	5q31.1	G	1.05		0.78	0.579	1.000
	*IL13*	rs1295686^f^	5q31.1	T	1.22		0.80	0.026	0.180
	*C11orf30-LRRC32*	rs7130588^g^	11q13.5	G	1.23		0.64	0.008	0.054
	*GSDMB*	rs2305480^f^	17q21.1	G	1.22		0.44	0.009	0.060
	*GSDMA*	rs3894194^f^	17q21.1	A	1.18	0.94	0.47	0.029	0.201
**AA** ^a^	**Gene**	**SNP**	**Locus**	**Effect Allele**	**OR**	**Rsq**	**Freq (%)**	**P-value** ^b,d^	**Adj P-value** ^e^
*(325 cases, 2342 controls)*	*IL1RL1*	rs10197862^f^	2q12.1	A	1.10	0.98	0.74	0.344	1.000
	*TSLP*	rs1837253^g^	5q22.1	C	1.11		0.28	0.283	1.000
	*RAD50*	rs2244012^f^	5q31.1	G	1.05		0.57	0.603	1.000
	*IL13*	rs1295686^f^	5q31.1	T	1.16		0.64	0.102	0.717
	*C11orf30-LRRC32*	rs7130588^g^	11q13.5	G	1.23		0.80	0.035	0.247
	*GSDMB*	rs2305480^f^	17q21.1	G	1.03		0.13	0.817	1.000
	*GSDMA*	rs3894194^f^	17q21.1	G	1.02	0.88	0.69	0.846	1.000
**Meta-analysis** ^a^	**Gene**	**SNP**	**Locus**	**Effect Allele**	**OR**	**I** ^**2**^		**P-value** ^c,d^	**Adj P-value** ^e^
*(710 cases, 7321 controls)*	***IL1RL1***	**rs10197862** ^**f**^	**2q12.1**	**A**	**1.23**	**70**		**0.006**	**0.042**
	*TSLP*	rs1837253^g^	5q22.1	C	1.01	45		0.910	1.000
	*RAD50*	rs2244012^f^	5q31.1	G	1.05	0		0.447	1.000
	***IL13***	**rs1295686** ^**f**^	**5q31.1**	**T**	**1.19**	**0**		**0.006**	**0.043**
	***C11orf30-LRRC32***	**rs7130588** ^**g**^	**11q13.5**	**G**	**1.23**	**0**		**0.001**	**0.005**
	*GSDMB*	rs2305480^f^	17q21.1	G	1.17	27		0.018	0.125
	*GSDMA*	rs3894194^f^	17q21.1	A	1.10	57		0.113	0.794

Abbreviations: *NHGRI-EBI* National Human Genome Research Institute - European Bioinformatics Institute; *SNP* single nucleotide polymorphism; *GWAS* genome-wide association study; *NHW* Non-Hispanic Whites; *AA* African American; *OR* odds ratio of the effect allele; *Rsq* estimation of imputation quality; *Freq* frequency of effect allele; *I*^*2*^ heterogeneity index; ^a^ Each population was run as an independent analysis, and then combined in the meta-analysis: ^b^ Logistic regression based on case control status; ^c^ Fixed effect meta-analysis weighted by inverse variance. ^d^ Adjusted for sex, genetic ancestry. ^e^ Bonferroni correction for seven tests, SNPs with adjusted p < 0.05 in Bold. ^f^ SNP is in the reported gene, ^g^ SNP is near the reported gene

Five SNPs associated with pediatric asthma in the TAGC GWAS were examined within the current GWAS results (Additional file 1: Table S1). Reported *p*-values were Bonferroni corrected for five tests, with a significance level of 0.05. Among the NHW population, the childhood asthma GWAS found association with TAGC pediatric asthma SNPs in *IL1RL1* (rs4988958, adjusted *p* = 0.002), *LINC01149* (rs2596464, adjusted *p* = 0.021), and near the *GSDMB* region (rs8069176, adjusted *p* = 0.041). The meta-analysis of NHW and AA subjects in out childhood asthma GWAS found association with *IL1RL1* (rs4988958, adjusted *p* = 0.035) and *LINC01149* (rs2596464, adjusted *p* = 0.017).

## Discussion

This analysis is the first to demonstrate that a self-reported history of childhood asthma is a valid method for defining a population that phenotypically and genetically represent asthmatic subjects among a cohort of adult smokers at risk for COPD. When compared to those who never had asthma, self-reported childhood asthmatics were younger at study enrollment, more likely to be of AA race, and had decreased lung function with greater odds of developing COPD. Three of seven established NHGRI-EBI asthma SNPs and three of five pediatric asthma SNPs from the TAGS GWAS were associated with self-reported childhood asthma, including SNPs in *IL1RL1*, *IL13*, near the *C11orf30-LRRC32* region, in *LINC01149*, and near *GSDMB*. Additionally, GWAS identified one variant in *KIAA1958* that was associated with childhood asthma among NHWs with genome-wide significance. Regions of interest were identified at near genome-wide significance among NHWs in *PDZD2*, among AAs in *FLJ12825* and *PHF14*, and on meta-analysis in *SCHIP1* and its read-through transcript *IQCJ-SCHIP1*.

There has been a growing interest in the risk of early life asthmatics for developing COPD, and thus ACO, in adulthood [15]. It has been proposed that in some childhood asthmatics, the risk for COPD is the result of the lungs never achieving their expected growth and development in early adulthood [11, 12, 16, 20, 52, 53]. Normal decline in lung function can lead to COPD since expected maximal FEV_1_ is never attained. ACO has been recognized as a distinct COPD subtype, with elevated risk for exacerbations, increased health-care burden, and different potential treatment modalities [54]. Research into the mechanisms of disease and genetic susceptibility for ACO subjects has been limited by the traditional exclusion of asthmatic subjects from large COPD studies [15]. Studies of COPD use spirometry as the accepted COPD definition; however, ACO research has been complicated by disagreement on a standard definition for asthma and therefore ACO [15, 34, 54–56]. COPD cohorts often include older adults and in many cases it is not possible to confirm early-life diagnosis of asthma by Global Initiative for Asthma (GINA) spirometry guidelines or by physician records [55, 57]. Once COPD has been diagnosed, it is difficult to use bronchodilator reactivity to define asthma, as this can also be a feature of COPD. Use of self-reported history of asthma has raised concern due to the theoretical risk of misclassification bias [58].

This study confirms that childhood asthmatics who smoke are at increased risk for developing low lung function and COPD as adults, when compared to smokers who never had asthma. This investigation supports self-reported diagnosis of asthma history as a valid method of identifying early-life asthmatics, and a population of ACO subjects, in large population cohorts at risk for COPD. This will be particularly important as researchers further examine the genetic epidemiology of ACO.

There have been a number of prior asthma GWAS conducted in populations of asthmatic subjects, beginning with the GABRIEL Consortium in 2007, which focused on populations of European ancestry, and continuing through the recent TAGC GWAS, a meta-analysis in ethnically diverse populations including 23,948 cases and 118,538 controls; the TAGS GWAS included a pediatric subgroup meta-analysis with 8976 cases and 18,399 controls [4, 24]. These asthma GWAS have used a range of asthma definitions, including physician diagnosis in GABRIEL, electronic medical record information in eMERGE, and physicians’ diagnosis and/or standardized questionnaires in the TAGC [4, 24, 47]. A number of prior GWAS have identified distinct genetic susceptibility for pediatric onset asthma, particularly implicating *ORLDM3*/*GSDMB*, *IL1RL1*, and *IL13*, which were all found to have association with childhood asthma in the current analysis [4, 5, 6, 7, 24, 59]. *C11orf30-LRRC3*, which also has association in this GWAS, has been associated with in pediatric asthma previously, though in prior GWAS the primary link with asthma has been in the setting of allergic disease [33, 60].

This study examined association with seven established asthma SNPs, selected as they were the only SNPs reported by more than one study among 85 studies in the NHGRI-EBI GWAS catalog, and five SNPs from the largest pediatric GWAS to date from the TAGC [2, 3, 24]. Five genes were found to be associated with self-reported childhood asthma in COPDGene: *IL1RL1*, *IL13*, *LINC01149*, near *GSDMB*, and in the *C11orf30-LRRC32* region. *IL1RL1* in the 2q12.1 region is thought to be involved in T-helper cell type 2 (TH2) inflammation including eosinophil activation [42, 44–46, 61–64]. *IL13* is an immunoregulatory cytokine produced by TH2 cells that is critical to the pathogenesis of allergic asthma, operating through mechanisms independent of IgE and eosinophils [65]. *GSDMB* in the established 17q21 childhood asthma risk region is potentially involved in pathogenesis via epithelial cell pyroptosis [4, 7, 41, 44, 47, 49, 50, 66]. In asthmatics, the *C11orf30-LRRC32* region is associated with total serum IgE levels [67]. We did not see evidence of association from three of the seven established NHGRI-EBI asthma SNPs, those found in *TSLP*, *RAD50*, and *GSDMA*. *TSLP* has a role in TH2 cell responses associated with inflammatory diseases including asthma and COPD [65]. *RAD50* has been implicated in allergic airway inflammation, potentially vial regulation of TH2 cytokine genes, though the exact mechanism remains to be elucidated [68]. *GSDMA* is along with *GSDMB* and *ORMDL3* part of the complex 17q21.1 locus, more research is needed to understand the specific interactions of variants in this region and their role in asthma pathobiology [69].

There was one novel genome-wide significant SNP associated with childhood asthma in the GWAS, rs59289606, an intron variant in the protein-coding gene *KIAA1958* at locus 9q32; this SNP is imputed with Rsq 0.38, which meets our criteria for imputation quality. *KIAA1958* has not been previously associated with disease pathogenesis [70]. In the AA population, variants of interest were found in *FLJ12825* and *PHF14*. *FLJ12825* is a non-coding RNA, and *PHF14* is a protein coding gene important in regulation of mesenchymal cell proliferation specific to lung fibrosis, that has also been described in newborn respiratory failure and newborn pulmonary hypertension [71, 72]. Meta-analysis identified a region of interest in *SCHIP1* and its read-through transcript *IQCJ-SCHIP1*. *SCHIP1* has been reported along with *IL12A* as a potential systemic sclerosis gene which functions in immune regulation via T cell activity [73].

### Limitations

This childhood asthma GWAS used a cohort of adult smokers at risk for COPD. It is underpowered relative to the large-scale asthma GWAS that have recently been published. However, our objective was not to run a novel asthma GWAS; rather, the goal of this investigation was to examine self-reported asthma in a population of adult smokers at risk for COPD. Ideally, we would have been able to compare this self-reported diagnosis of childhood asthma to a gold-standard diagnosis, such as childhood spirometry or medical records, but this primary data in this cohort of older adults is not available. Notably, when compared to clinical diagnosis, questionnaire reported asthma history has been shown to have relatively good agreement and reliability over time [74–76]. It would have additionally been valuable to use medical records to assess asthma treatment and control; however, we note that many of these subjects had childhood asthma prior to the widespread use of inhaled corticosteroids in the US. Additionally, asthma treatment will not impact the patients’ genotype, so the objectivity of that measure is maintained. Since COPDGene includes smokers, we were not able to assess COPD risk among non-smokers; given that this investigation was focused on a population with a high COPD risk, including only smokers is reasonable.

The assessed list of known asthma variants were selected from 600 asthma associations published in the NHGRI-EBI GWAS catalog, where only eight independent SNPs were reported in more than one study. An additional set of five pediatric asthma GWAS SNPs from the TAGC was also examined. We acknowledge that alternative lists of established asthma SNPs could be proposed. It is notable that the established asthma variant list was largely composed of SNPs from European American or NHW populations, and thus the lack of associations in the AA population is not unexpected. This also explains why the combined NHW and AA meta-analysis did not improve power to detect associations for some of the SNPs. It would have been desirable to include other populations with alternative genetic heritage, but COPDGene is limited to NHW and AA subjects.

## Conclusions

Self-report of childhood asthma in adult smokers from COPDGene identifies a meaningful population who demonstrate the demographic, clinical, and genetic characteristics known to associate with childhood asthma. Compared to subjects who never had asthma, self-reported childhood asthmatics were younger, more likely to be of AA race, and had increased odds of COPD. We showed associations with known asthma loci in *IL1RL1*, *IL13*, *LINC01149*, near *GSDMB*, and in the *C11orf30-LRRC32* region. This GWAS identified a new variant in *KIAA1958* associated with childhood asthma in NHWs. This study will enhance future genetic epidemiology research in early-life asthmatics at risk for COPD and therefore ACO, by establishing the validity of self-reported asthma in the research setting. This study emphasizes that clinicians need to consider childhood asthma when assessing patients at increased risk for COPD, and that patient self-report is a reliable method of defining ACO.

## Additional files

Additional file 1: **Figure S1.** Subject classification. **Figure S2.** QQ-plot and Manhattan plot of childhood asthma GWAS SNPs in non-Hispanic Whites. **Figure S3.** QQ-plot and Manhattan plot of childhood asthma GWAS SNPs in African Americans. **Figure S4.** QQ-plot and Manhattan plot of childhood asthma GWAS SNPs from meta-analysis of NHW and AA subjects. **Figure S5.** LocusZoom Plots of childhood asthma non-Hispanic white GWAS variants in *PDZD2*. **Figure S6.** LocusZoom Plots of childhood asthma African American GWAS variants in regions of interest in *FLJ12825* and *PHF14*. **Figure S7.** In non-Hispanic whites the childhood asthma GWAS found association with the known asthma genes *IL1RL1* (rs10197862, adj *p* = 0.011) and *GSDMB* (rs2305480, adj *p* = 0.060). **Figure S8.** Meta-analysis of NHW and AA subjects in this study found association with a known asthma variants in IL13 (rs1295686) and near the *C11orf30-LRRC32* region (rs7130588). **Table S1.** Associations with TAGC Pediatric Asthma GWAS SNPs. (PDF 977 kb)

## Abbreviations

AA: African American
ACO: Asthma-COPD overlap
CI: Confidence interval
COPD: Chronic obstructive pulmonary disease
FEV_1_: Forced expiratory volume in the first second
FVC: Forced vital capacity
GWAS: Genome-Wide Association Study
LD: Linkage disequilibrium
MAF: Minor allele frequency
NHGRI-EBI: National Human Genome Research Institute - European Bioinformatics Institute
NHW: Non-Hispanic white
OR: Odds ratio
PC: Principal components
QQ: Quantile-Quantile
SE: Standard error
SNP: Single nucleotide polymorphism
TAGC: Trans-National Asthma Genetic Consortium
TH2: T-helper cell type 2
