# Supplementary material for: Childhood asthma is associated with COPD and known asthma variants in COPDGene: a genome-wide association study
Source: Respir Res. 2018 Oct 29;19:209. doi: 10.1186/s12931-018-0890-0 (PMC6206739; doi:10.1186/s12931-018-0890-0)
Supplement: Supplementary file 1 — Figure S1. Subject classification. Figure S2. QQ-plot and Manhattan plot of childhood asthma GWAS SNPs in non-Hispanic Whites. Figure S3. QQ-plot and Manhattan plot of childhood asthma GWAS SNPs in African Americans. Figure S4. QQ-plot and Manhattan plot of childhood asthma GWAS SNPs from meta-analysis of NHW and AA subjects. Figure S5. LocusZoom Plots of childhood asthma non-Hispanic white GWAS variants in PDZD2. Figure S6. LocusZoom Plots of childhood asthma African American GWAS variants in regions of interest in FLJ12825 and PHF14. Figure S7. In non-Hispanic whites the childhood asthma GWAS found association with the known asthma genes IL1RL1 (rs10197862, adj p = 0.011) and GSDMB (rs2305480, adj p = 0.060). Figure S8. Meta-analysis of NHW and AA subjects in this study found association with a known asthma variants in IL13 (rs1295686) and near the C11orf30-LRRC32 region (rs7130588). Table S1. Associations with TAGC Pediatric Asthma GWAS SNPs. (PDF 977 kb) [file 12931_2018_890_MOESM1_ESM.pdf]

ADDITIONAL FILE 1

Table S1: Associations with TAGC Pediatric Asthma GWAS SNPs

**NHW**[a]	**Gene**	**SNP**	**Locus**	**Allele**	**OR**	**P-value**[b,d]	**Adj P**[e]
(385 cases, 4979 controls)	***IL1RL1***	**rs4988958**[f]	**2q12.1**	**T**	**1.33**	**0.0004**	**0.002**
	IL13	rs1295685[f]	5q31.1	A	1.18	0.073	0.363
	LINC01149	**rs2596464**[f]	**6p21.33**	**C**	**1.24**	**0.004**	**0.021**
	IL33	rs12551256[f]	9p24.1	G	1.08	0.284	1.000
	GSDMB	**rs8069176**[g]	**17q12-21**	**G**	**1.22**	**0.008**	**0.041**
**AA**[a]	**Gene**	**SNP**	**Locus**	**Allele**	**OR**	**P-value**[b,d]	**Adj P**[e]
(325 cases, 2342 controls)	*IL1RL1*	rs4988958[f]	2q12.1	C	1.00	0.991	1.000
	IL13	rs1295685[f]	5q31.1	A	1.12	0.516	1.000
	LINC01149	rs2596464[f]	6p21.33	C	1.10	0.246	1.000
	IL33	rs12551256[f]	9p24.1	A	1.02	0.846	1.000
	GSDMB	rs8069176[g]	17q12-21	A	1.02	0.824	1.000
**Meta-analysis**[a]	**Gene**	**SNP**	**Locus**	**Allele**	**OR**	**P-value**[c,d]	**Adj P**[e]
(710 cases, 7321 controls)	***IL1RL1***	**rs4988958**[f]	**2q12.1**	**T**	**1.18**	**0.007**	**0.035**
	IL13	rs1295685[f]	5q31.1	A	1.16	0.059	0.296
	LINC01149	**rs2596464**[f]	**6p21.33**	**C**	**1.18**	**0.003**	**0.017**
	IL33	rs12551256[f]	9p24.1	G	1.05	0.437	1.000
	GSDMB	rs8069176[g]	17q12-21	G	1.12	0.058	0.291

Abbreviations: *TAGC* Trans-National Asthma Genetic Consortium, *GWAS* genome-wide association study; *SNP* single nucleotide polymorphism; *NHW* Non-Hispanic Whites; *AA* African American; *OR* odds ratio of the effect allele; [a] Each population was run as an independent analysis, and then combined in the meta-analysis: [b] Logistic regression based on case control status; [c] Fixed effect meta-analysis weighted by inverse variance. [d] Adjusted for sex, genetic ancestry. [e] Bonferroni correction for five tests, SNPs with adjusted $p<0.05$ in **Bold**. [f] SNP is in the reported gene, [g] SNP is near the reported gene.

Figure S1

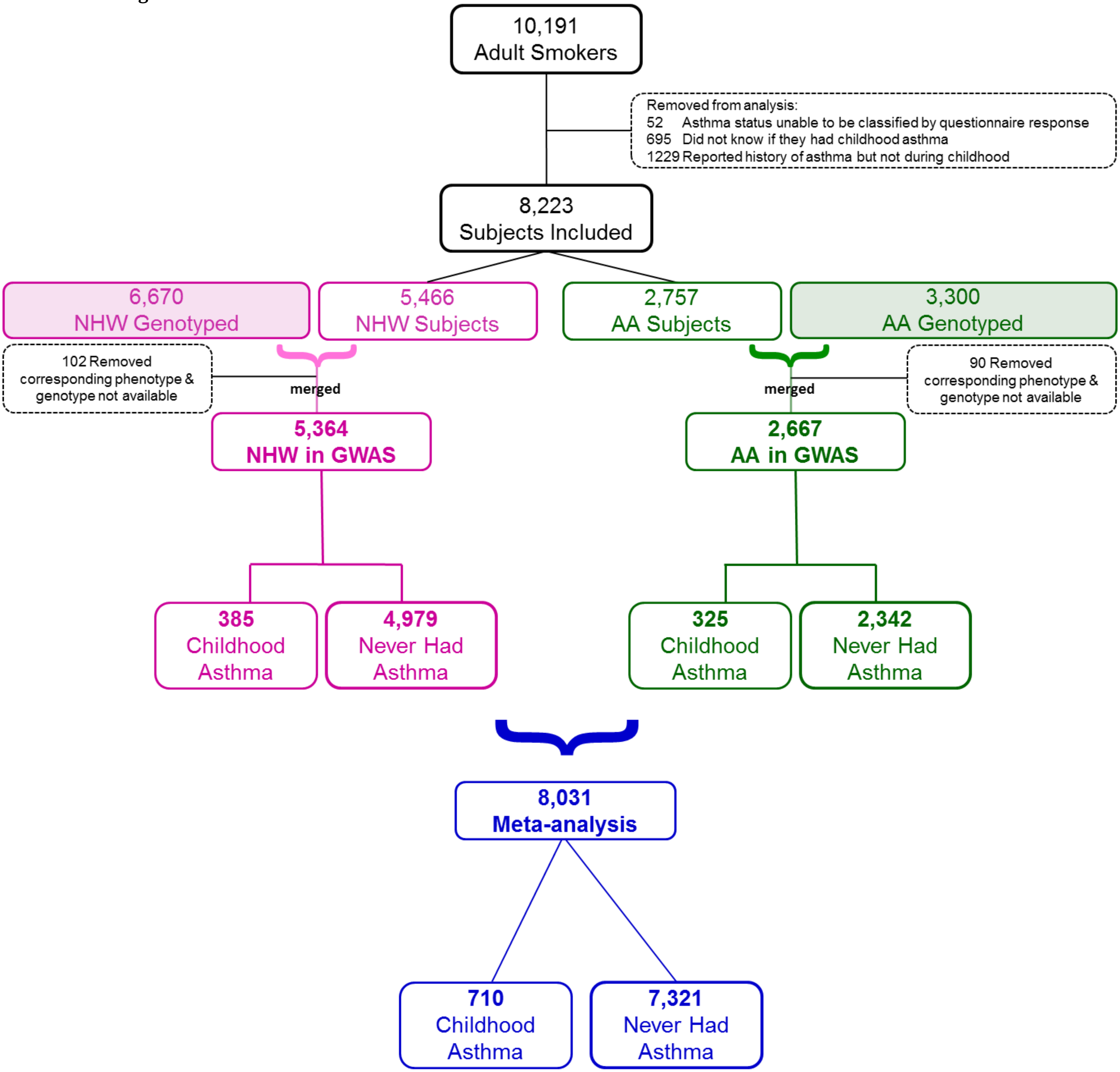

Figure S1: Subject classification. Abbreviations: *NHW* non-Hispanic White; *AA* African American.

Figure S2

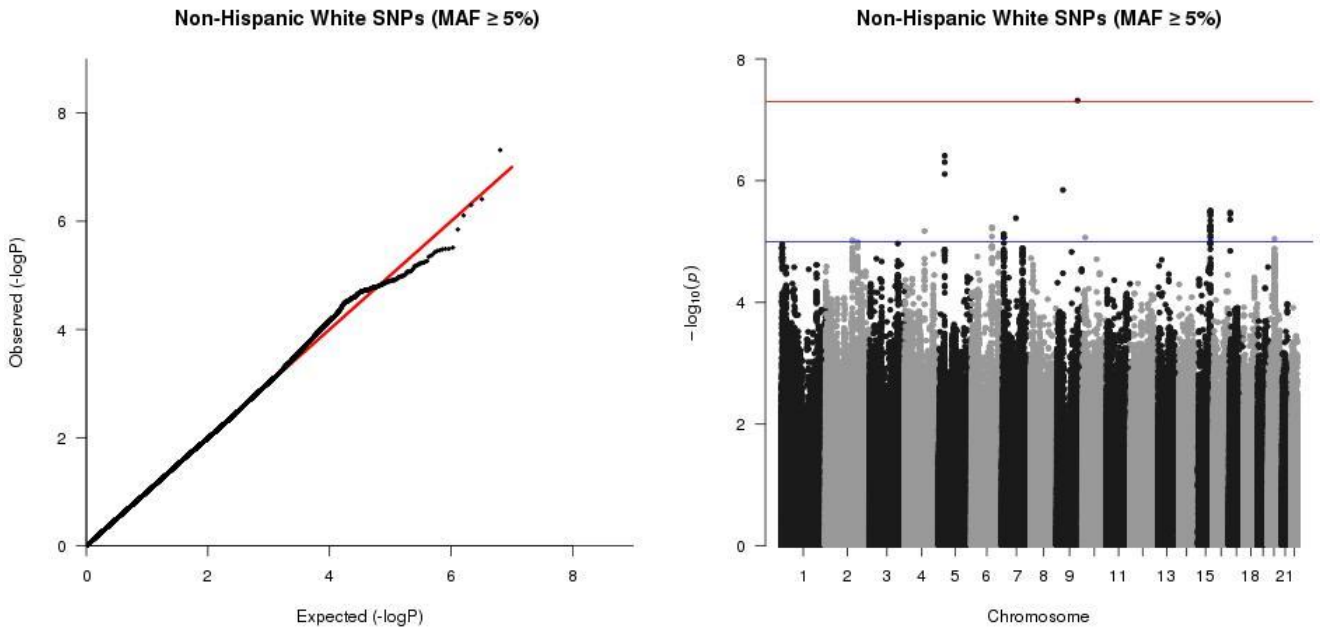

Figure S2: QQ-plot and Manhattan plot of childhood asthma GWAS SNPs in non-Hispanic Whites. Abbreviations: *SNP* single nucleotide polymorphism; *MAF* minor allele frequency.

Figure S3

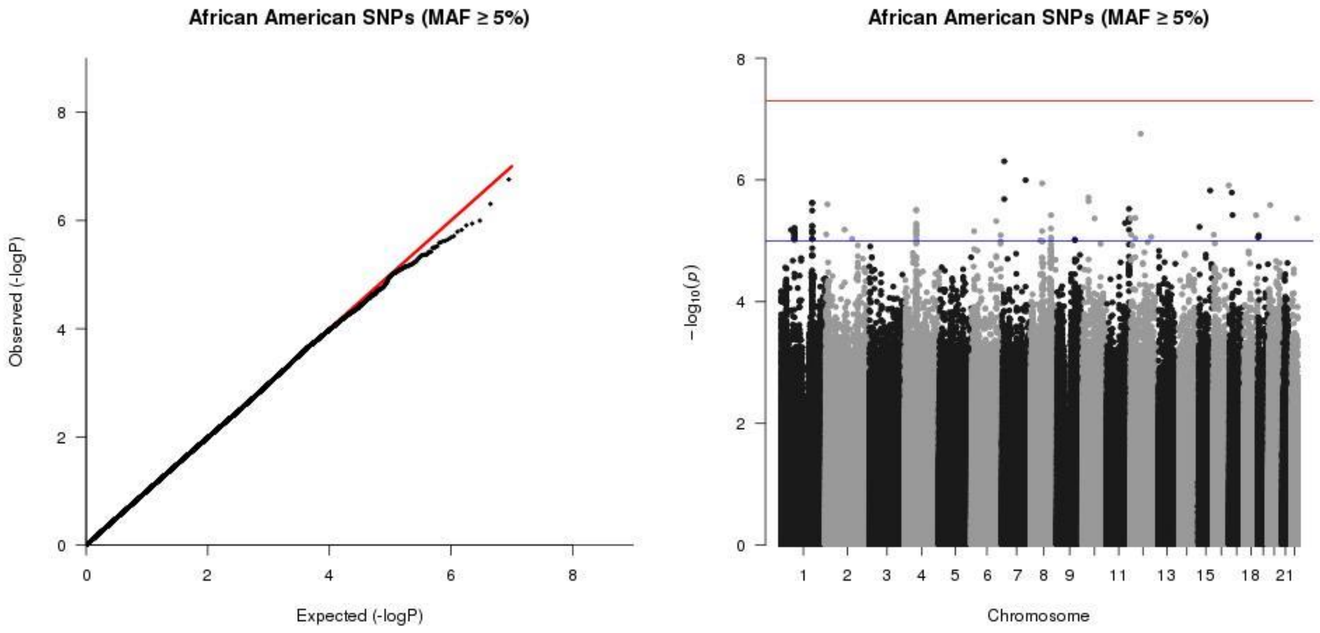

Figure S3: QQ-plot and Manhattan plot of childhood asthma GWAS SNPs in African Americans. Abbreviations: *SNP* single nucleotide polymorphism; *MAF* minor allele frequency.

Figure S4

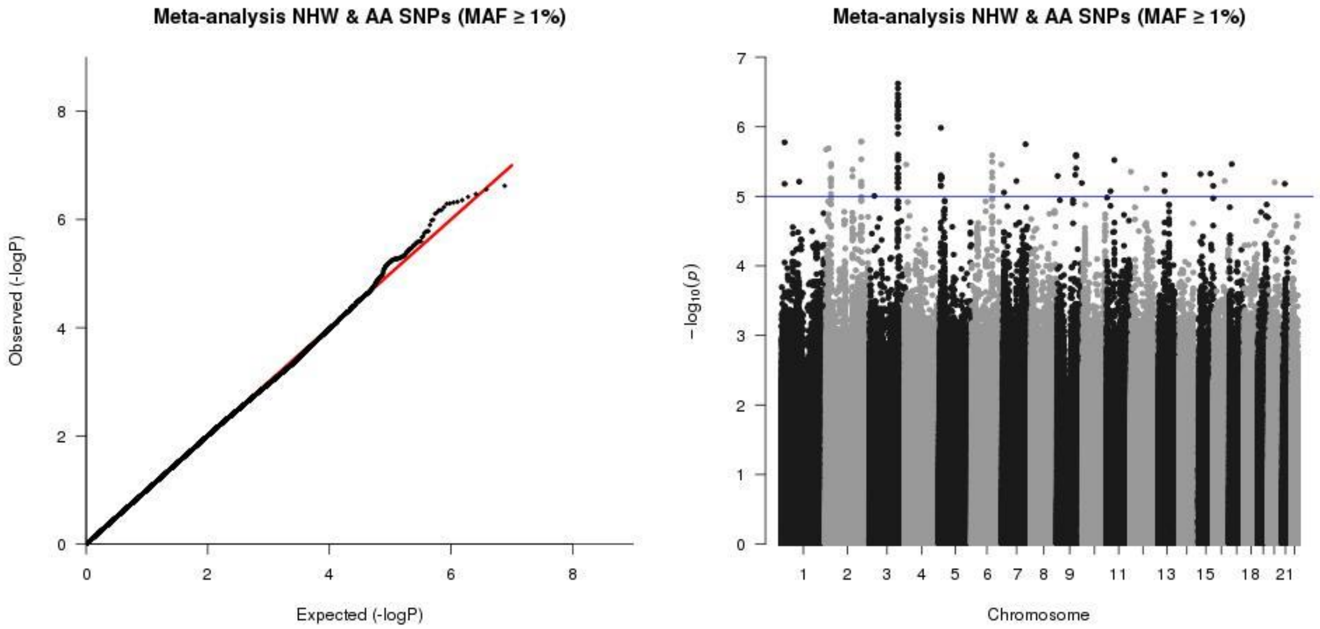

Figure S4: QQ-plot and Manhattan plot of childhood asthma GWAS SNPs from meta-analysis of NHW and AA subjects. Abbreviations: *NHW* non-Hispanic White, *AA* African American, *SNP* single nucleotide polymorphism; *MAF* minor allele frequency.

Figure S5

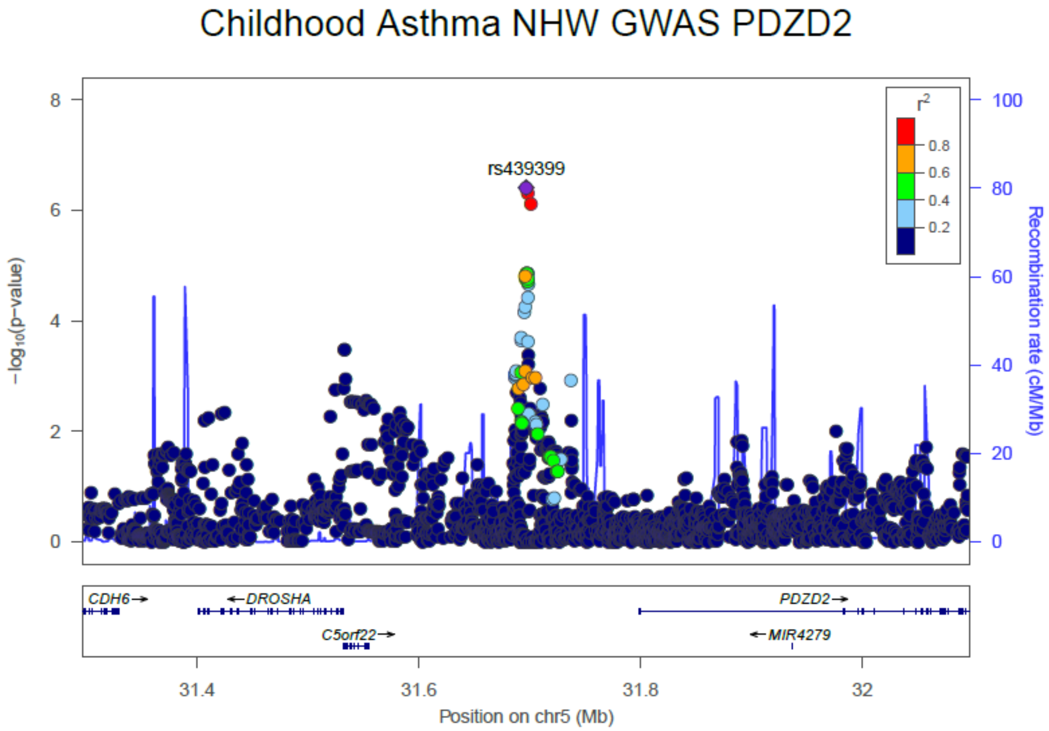

Figure S5: LocusZoom Plots of childhood asthma non-Hispanic white GWAS variants in *PDZD2*.

Figure S6

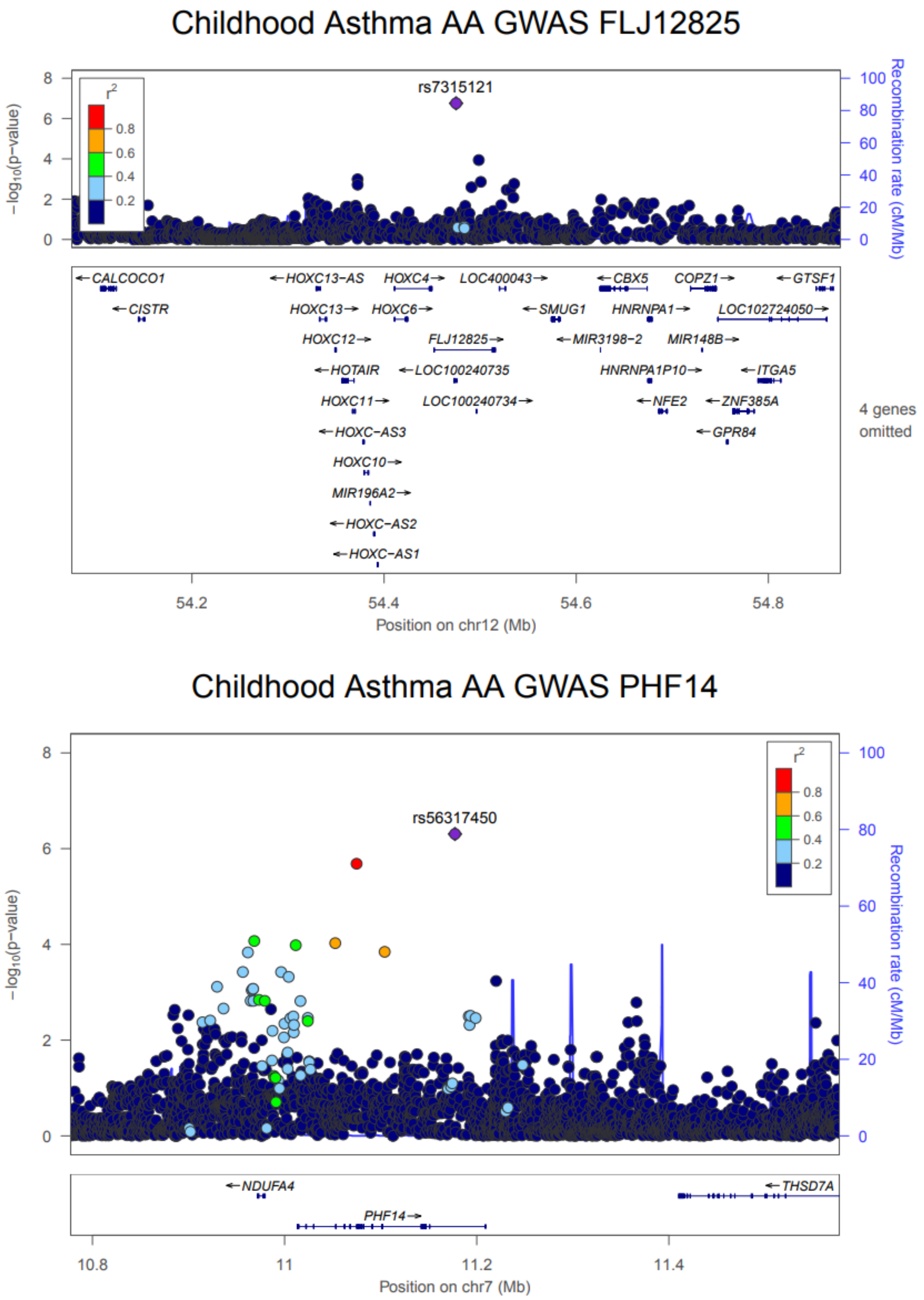

Figure S6: LocusZoom Plots of childhood asthma African American GWAS variants in regions of interest in *FLJ12825* and *PHF14*.

Figure S7

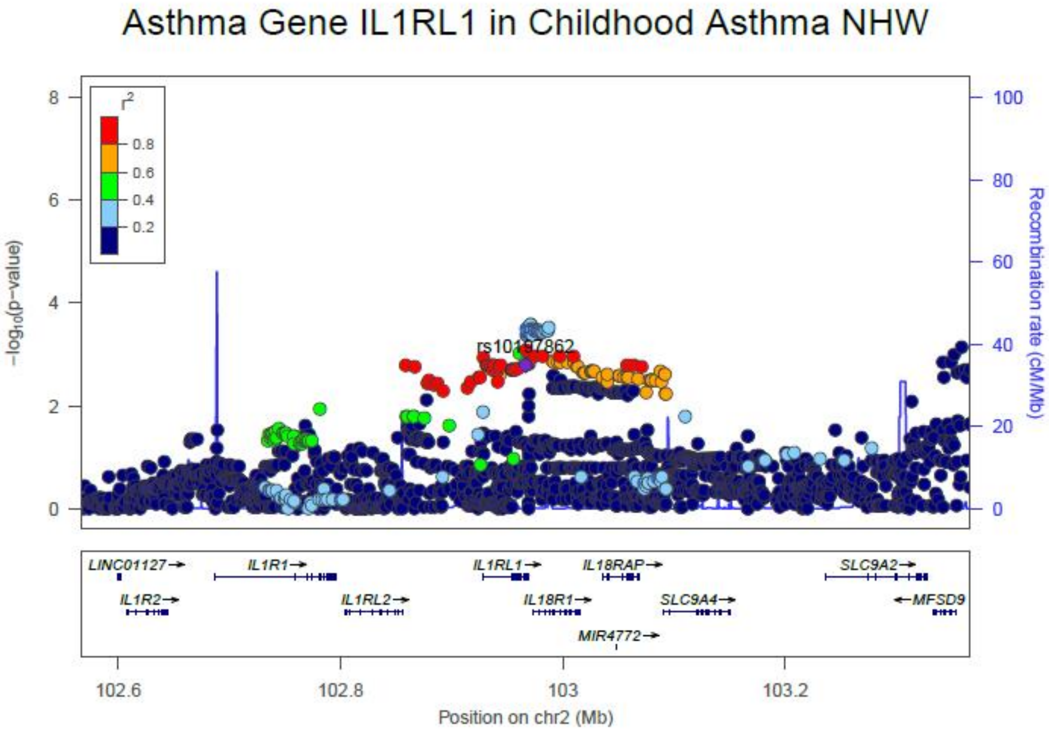

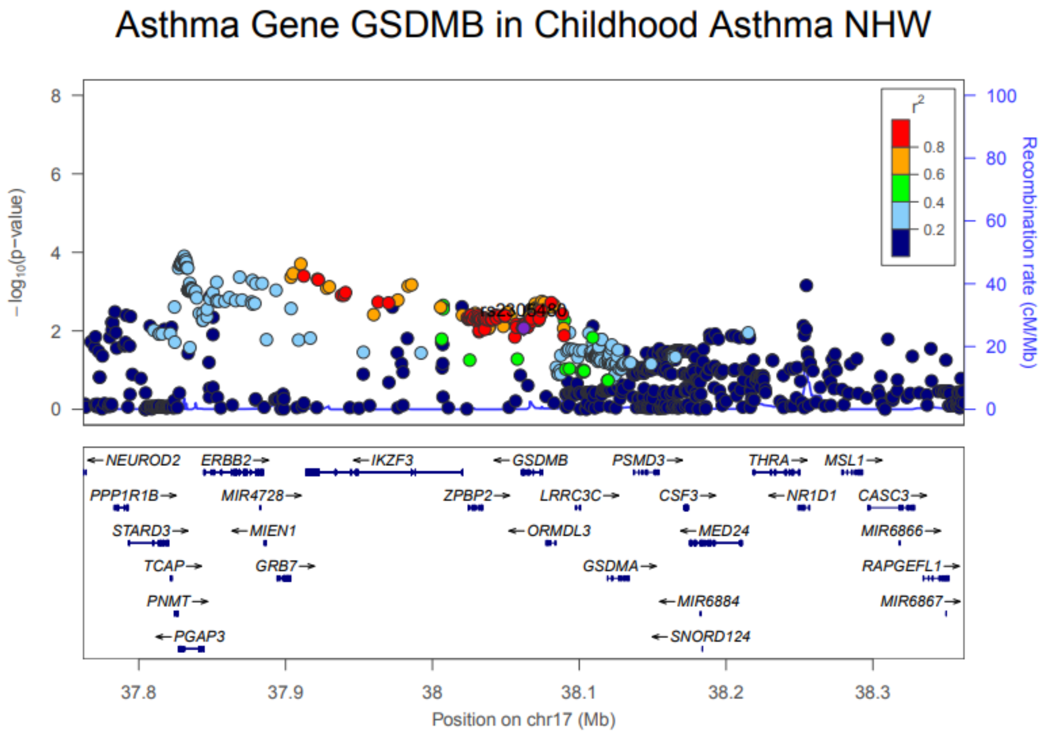

Figure S7: In non-Hispanic whites the childhood asthma GWAS found association with the known asthma genes *IL1RL1* (rs10197862, adj p = 0.011) and *GSDMB* (rs2305480, adj p = 0.060).

Figure S8

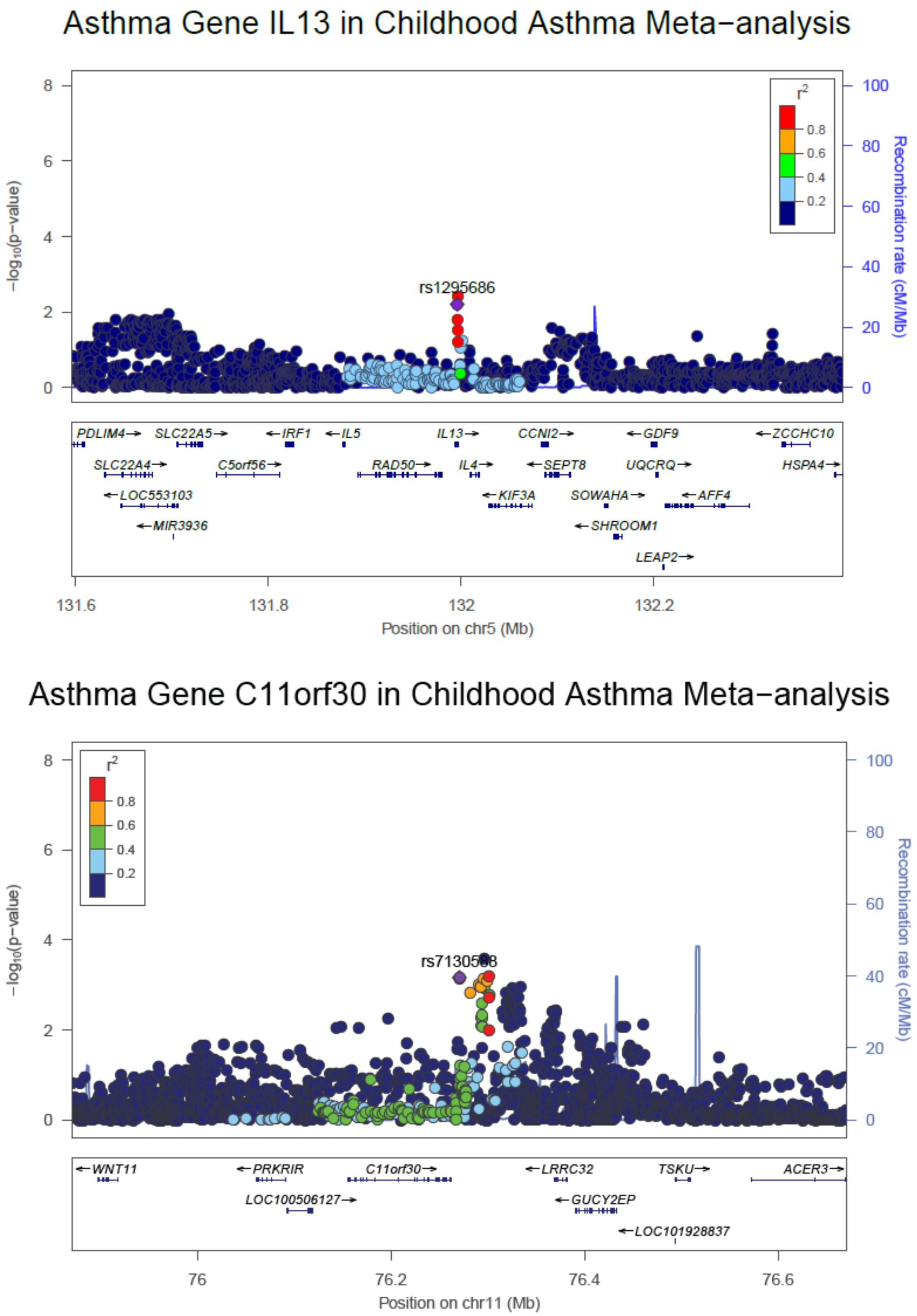

Figure S8: Meta-analysis of NHW and AA subjects in this study found association with a known asthma variants in IL13 (rs1295686) and near the *C11orf30-LRRC32* region (rs7130588).
